# In Silico Analysis: Molecular Characterization and Evolutionary Study of *CLCN* Gene Family in Buffalo

**DOI:** 10.3390/genes15091163

**Published:** 2024-09-03

**Authors:** Yiheng Fu, Muhammad Farhan Khan, Yingqi Wang, Shakeela Parveen, Mehwish Sultana, Qingyou Liu, Laiba Shafique

**Affiliations:** 1 State Key Laboratory for Conservation and Utilization of Subtropical Agro-Bioresources, College of Animal Science and Technology, Guangxi University, Nanning 530004, China; 2118401001@st.gxu.edu.cn; 2 Guangxi Key Laboratory of Beibu Gulf Marine Biodiversity Conservation, Beibu Gulf University, Qinzhou 535011, China; farhankhanbgu@gmail.com; 3 Department of Chemistry, Gomal University, Dera Ismail Khan 29050, Pakistan; 4 Guangdong Provincial Key Laboratory of Animal Molecular Design and Precise Breeding, School of Life Science and Engineering, Foshan University, Foshan 528225, China; qishao0410@163.com; 5 Department of Zoology, Government Sadiq College Women University, Bahawalpur, Punjab 63100, Pakistan; drshakeela@gscwu.edu.pk (S.P.); mehwishsultana35@gmail.com (M.S.)

**Keywords:** buffalo, breakpoint, *CLCN* gene, duplication, Ka/Ks ratio

## Abstract

Chloride channels (ClCs) have received global interest due to their significant role in the regulation of ion homeostasis, fluid transport, and electrical excitability of tissues and organs in different mammals and contributing to various functions, such as neuronal signaling, muscle contraction, and regulating the electrolytes’ balance in kidneys and other organs. In order to define the chloride voltage-gated channel (CLCN) gene family in buffalo, this study used in silico analyses to examine physicochemical properties, evolutionary patterns, and genome-wide identification. We identified eight *CLCN* genes in buffalo. The ProtParam tool analysis identified a number of important physicochemical properties of these proteins, including hydrophilicity, thermostability, in vitro instability, and basic nature. Based on their evolutionary relationships, a phylogenetic analysis divided the eight discovered genes into three subfamilies. Furthermore, a gene structure analysis, motif patterns, and conserved domains using TBtool demonstrated the significant conservation of this gene family among selected species over the course of evolution. A comparative amino acid analysis using ClustalW revealed similarities and differences between buffalo and cattle CLCN proteins. Three duplicated gene pairs were identified, all of which were segmental duplications except for *CLCN4-CLCN5*, which was a tandem duplication in buffalo. For each gene pair, the Ka/Ks test ratio findings showed that none of the ratios was more than one, indicating that these proteins were likely subject to positive selection. A synteny analysis confirmed a conserved pattern of genomic blocks between buffalo and cattle. Transcriptional control in cells relies on the binding of transcription factors to specific sites in the genome. The number of transcription factor binding sites (TFBSs) was higher in cattle compared to buffalo. Five main recombination breakpoints were identified at various places in the recombination analysis. The outcomes of our study provide new knowledge about the *CLCN* gene family in buffalo and open the door for further research on candidate genes in vertebrates through genome-wide studies.

## 1. Introduction

Genetic variations that affect economically significant production qualities, differential response to diseases, and positive host responses to vaccinations is the ultimate objective of bovine genomics. With careful genetic selection, it is anticipated that this understanding will improve these characteristics [1]. Animals of high economic value include buffaloes (*Bubalus bubalis*), particularly in Asian regions. Among agricultural animals, buffalo is becoming more and more popular as a source of milk, meat, and draft power. In comparison to cattle, the buffalo has received far less attention and has been ignored despite its significant contributions as an economically important animal. There are two types of domestic buffalo: river buffalo and swamp buffalo. Swamp buffalo are utilized for labor in crop fields and the production of meat, while river buffalo are mostly raised for their milk and dairy products [2]. Swamp buffalo are mostly raised in southeast Asia, southeast China, Thailand, and southern China, while river buffalo are generally found in southwestern Asia, Egypt, India, and southern Europe [3]. More than 65% of the world buffalo population is found in China, India, and Pakistan.

Understanding the genetic basis of many biological processes, including growth, development, reproduction, and disease susceptibility, is made possible by genomics. Functional genomics can solve the problem of interpreting the biology of animals regarding their phenotypes, with the ability to identify which genes are relevant to certain characteristics or diseases [4]. Chloride channels that control the transport of chloride ions into or out of cells with an aim of balancing the body’s internal environment are proteins associated with the *CLCN* gene, which is composed of gene families from *CLCN* genes [5]. These genes play roles in several physiological processes in animals, including metabolic concerns, binding and transport, and acting as biochemical indicators [6].

CLC anion transporters are constitutively expressed in all phyla and form eight gene family members in mammals [7]. In the recent past, chloride channels or ClCs have been receiving interest across the globe because of their molecular variance across mammalian species, their tissue (organ) distribution, and their affiliated pathophysiology with certain human diseases. The gene family of mammal *ClCs* has been molecularly as well as functionally described [8]. Specifically, VGCIC for ClC-1, the *CLCN1* gene, was originally cloned from rat skeletal muscles [9] and exists within homodimers [10,11]. CLCN2 is critical for mammalian cells because it contributes to the volume-regulated chloride current (ICl.vol). Microorganisms are used to create the volume-regulated chloride current (ICl.vol) with the intention of controlling a series of functions, such as electrical functions, the volume of a cell, and balance of the cell pH [12]. As a chloride channel protein, it ensures the high endothelial venule (HEV) construction is intact when immune reactions are taking place. Some of its mutation might lead to the loss of vascular integrity and spontaneous bleeding in lymph nodes [13]. Hence, it is concluded that the *CLCN2* gene is involved in a multifunctional protein that is critical for immune response regulation, cellular homeostasis, and viral infection processes in mammals.

*CLCN3* is important for development and metabolism regulation in mammals, while mutations of this gene are related to development disorders and intellectual disabilities [14]. Functionally, *CLCN3* is considered to be instrumental and indispensable in the control of inflammation of adipose tissue and obesity in mammals, and therefore, it has been useful in the therapeutic treatment of type 2 diabetic patients and obesity [15]. Encoding the vesicular 2Cl−/H+ exchanger, ClC-4 is translated from the gene *CLCN4*, which is implicated with neurological disorders, including epilepsy and neurodevelopmental disability. It is vital in numerous processes in mammals [16,17]. *CLCN4* knockout mice studies revealed its role in ASD, affecting dendritic outgrowth and synapse remodeling, with consequences for drug development and perceptions into X-linked gene regulation [18].

CLCN5 acts during neurodevelopment and for the proper functioning of lysosomes; a mutation in this gene would result in a loss of function that leads to variant late-infantile neuronal ceroid lipofuscinosis [19,20]. Apart from that, CLCN5 has been implicated in the regulation of autophagy and endocytosis, and it also plays a role in the regulation cysteine palmitoyl thioesterase activity via sorting receptors. This modulation determines the regulation of both cardio metabolism and neuronal functioning [21,22]. Therefore, CLCN5 is a complex protein that plays specific roles in lysosomal metabolism and neuronal as well as cardiac health in mammals [23].

In mammals, directional DNA transport is very crucial during the formation of lungs and neurons. Pressure regulation, lung development, and neurons are directly affected by it. Therefore, based on the presented results, it could be assumed that *CLCN6* might be suitable for tumor-specific therapy as it is overexpressed in carcinomas [24,25]. There is enormous reliance on a protein called CLCN6, which is a member of the chloride family of proteins for the proper functioning of nerves, muscles, and cell ion balance in mammals.

Clathrin light chains and chromosome 6 are discussed in the context of protein diversity and genome research. In addition, the interconnection between sirtuin 6 and the protein regulation of lifespan is highlighted [26,27], particularly the CLCN7 chloride channel residing in the late endosomes and lysosomes. Genetic deficiency of Ostm1 results in the deficiency of a complex that in turn leads to lysosomal and protein stability. Certain polymorphisms in the gene encoding CLCN7 protein disrupt osteoclast functionality and bone resorption, leading to the development of severe osteopetrosis. Here, the basic level of the protein is directly related to the rate of lysosomal storage and the development of neurodegenerative diseases and proper functioning of cells. For this, the CLCN7-Ostm1 interaction is crucial for maintaining protein levels, impacting lysosomal storage, neurodegeneration, and overall cellular function [28]. CLC-k channels are located at the apical site; more specifically, these channels are primarily present in the kidneys. Focusing on the zebrafish and human models of the CLC-k channels, the importance of apical chloride reabsorption for maintaining chloride balance has been discussed [29].

Of several mammal species, the buffalo (*Bubalus bubalis*), an agriculturally important animal with high economic potential, ranks high due to the relevance of the *CLCN* gene family. The chloride channel is required for a number of physiological processes in buffalo, and one of them is for the secretion of fluid from mammary glands required for milk production. The improvement in strategies aimed at enhancing the health and production of the buffalo, such as strengthening disease resistance, electrolyte balance, and the level of reproduction, could benefit from *CLCN* gene activities. The present study was designed to identify buffalo’s *CLCN* gene family members; to perform a phylogenetic analysis, gene structure analysis, and recombination analysis; to analyze their physiochemical properties, gene duplication, and transcription binding sites; and to perform a comparative amino acid analysis in comparison with cattle.

## 2. Material and Method

### 2.1. Identification of the CLCN Gene Family

The *CLCN* gene family sequences of typical farm animals, such as buffalo and cattle, were obtained from online genome databases from NCBI https://www.ncbi.nlm.nih.gov/ (accessed on 30 June 2024), using the accession numbers indicated in Supplementary Table S1. The conserved HMG-box domain protein, a DNA binding protein, was profiled using the Hidden Markov Model (HMM) profile from the Pfam database in order to discover potential *CLCN* genes in various livestock species. A local BLASTP program was used to explore the predicted protein-coding variations, with E values set at 105 [30].

### 2.2. Phylogenetic and Multiple Sequence Alignment Analysis

The NCBI database was utilized to obtain the *CLCN* gene sequences for the following species: Murrah buffalo, swamp buffalo, Mediterranean buffalo, *Bos indicus*, *Bos taurus*, *Bos indicus x Bos taurus*, *Sus scrofa*, *Camelus bactrianus*, *Ovis aries*, *and Capra hircus*. ClustalW was utilized to align gene sequences from every species using MEGA7 software. After that, a neighbor-joining (NJ) phylogenetic tree was built in MEGA11 using 1000 replicates for the bootstrap value. Then, by utilizing ITOL and a single aligned file, the tree was constructed and presented [31]. In order to detect sequence alterations or insertions and deletions or indels, the *CLCN* genes of cattle and buffalo were also aligned using Multiple Align Show https://www.bioinformatics.org/sms/multi_align.html (accessed on 6 July 2024).

### 2.3. Physicochemical Properties and Structure Analysis

The buffalo CLCN proteins were physiochemically characterized using the ProtParam program, which yielded information on the molecular weight (MW), aliphatic index (AI), instability index (II), number of amino acids (A.A.), and isoelectric point (pI). Furthermore, conserved protein motifs in the buffalo CLCN proteins were identified using the MEME suite, which could identify up to 10 MEME motifs. The conserved domains of the buffalo CLCN proteins were also verified using the NCBI CDD database https://www.ncbi.nlm.nih.gov/Structure/cdd/wrpsb.cgi (accessed on 10 July 2024) [32]. Using the web servers Cello-life http://cello.life.nctu.edu.tw/ (accessed on 12 July 2024) and Wolf Psort https://wolfpsort.hgc.jp/ (accessed on 13 July 2024), the subcellular localization of the buffalo CLCN proteins was predicted [33].

### 2.4. Gene Duplication and Chromosomal Location of CLCN Gene Family

Using the buffalo whole-genome dataset, the chromosomal length and location of the *CLCN* genes were ascertained. The MCScanX tool was used to map the precise positions of the genes on the chromosomes based on the genome annotation file. The pairwise synonymous substitutions per synonymous site (Ks) and pairwise nonsynonymous substitutions per nonsynonymous site (Ka) were computed using the online Ka/Ks calculation tool http://services.cbu.uib.no/tools/kaks (accessed on 18 July 2024) [34].

### 2.5. Three-Dimensional Structure Analysis

The amino acid sequences of all the CLCN proteins of buffalo and cattle were submitted to online server Phyre2 http://www.sbg.bio.ic.ac.uk/phyre2 (accessed on 19 July 2024) to construct a three-dimensional structure of each CLCN protein, and the secondary structure features, fold recognition, and homology modeling were evaluated [30].

### 2.6. Scan Prosite Analysis and Analysis of Syntenic Relationships

An online tool called Scan Prosite was utilized to evaluate the structural and functional variations within a domain [35]. The Prosite motif library was searched by uploading a protein sequence using the web application https://prosite.expasy.org/scanprosite (accessed on 21 July 2024) [36,37]. Using the genome visualization tool Circoletto, comparative genomic synteny was used to investigate the link between the CLCN genes of buffalo and cattle [38].

### 2.7. Retrieval and Identification of Transcription Factor Binding Sites (TFBSs)

The genomic data were uploaded to the TFBIND program https://tfbind.hgc.jp/ (accessed on 23 July 2024), which searches the 100 bp region upstream of the highly predicted location for potential transcription factor binding sites using the TRANSFAC R.3.4 weight matrix [39]. One repressor site (YY1) and four transcriptional promoting binding sites (GATA, TATA, STAT, and OCT-1) were investigated. Instructions for making a protein that binds to particular DNA sequences and aids in controlling the activity of other genes were provided by the GATA transcription factor [40].

### 2.8. Analysis of Recombination Breakpoints in the CLCN Gene Family

Using the Genetic Algorithm Recombination Detection (GARD) program, recombination breakpoints in numerous sequence-aligned buffalo *CLCN* genes were identified [41]. The objective of this approach is to collect evidence of segment-specific phylogenies. If the maximum number of breakpoints (B, which may also be deduced) is known, it looks for B or fewer breakpoints in the alignment. Using a maximum likelihood model fit for each segment, the technique derives phylogenies for each possible non-recombinant segment [42] and assesses the fit using pertinent data criteria, like the Akaike information criteria (AIC).

## 3. Results

### 3.1. Phylogenetic Analysis and Multiple Sequence Analysis

**Figure 1:**
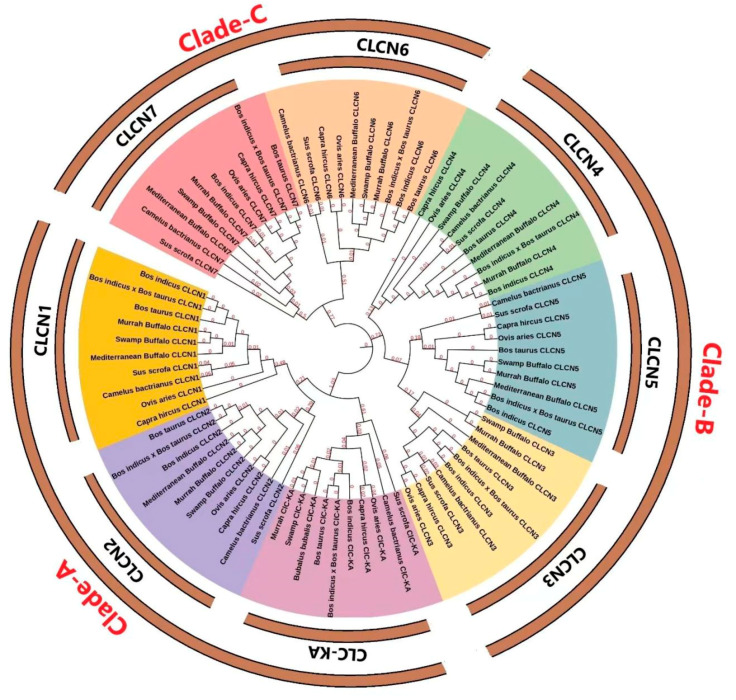
Phylogenetic analysis of *CLCN* gene family in selected species with three clades (A, B, and C). Clade-A subdivided into *CLCN1*, *CLCN2*, and *CLC-KA*; Clade-B subdivided into *CLCN4*, *CLCN5*, and *CLCN6*; and Clade-C subdivided into *CLCN6* and *CLCN7*.

The evolutionary history of the *CLCN* gene family was ascertained using molecular phylogenetic analysis utilizing the maximum likelihood method, whereby each node’s bootstrap consensus values were provided. Based on homologous gene sequences, 80 amino acid sequences from the following species were analyzed: Murrah buffalo, swamp buffalo, Mediterranean buffalo, *Bos indicus*, *Bos taurus*, *Bos indicus x Bos taurus*, *Sus scrofa*, *Camelus bactrianus*, *Ovis aries*, and *Capra hircus*. These sequences were then grouped into three groups: Clade-A, Clade-B, and Clade-C (Figure 1). The results of the phylogenetic analysis indicated that there are more sequence similarities between the buffalo *CLCN* gene family and the Murrah buffalo and swamp buffalo. Based on the data presented, many alignments demonstrated the commonalities, discrepancies, and indels between buffalo and cattle (Supplementary Figures S1–S8).

### 3.2. Identification and Physicochemical Properties of CLCN Gene Family in Buffalo and Cattle

In this study, the data of eight *CLCN* genes were collected from the genomes of cattle and buffalo by using the NCBI database. The physicochemical characteristics of these genes were examined, including their molecular weight (MW), aliphatic index (AI), instability index (II), isoelectric point (pI), and grand average of hydropathicity (GRAVY) (Table 1a). All the buffalo CLCN proteins have lengths ranging from 687 to 989 amino acid residues; in cattle, the same range applies. Cattle’s CLCN proteins have a molecular weight range of 75,172.78 D to 108,857.33 D, whereas buffalo varies from 75,182.79 D to 109,021.59 D. In buffalo, the isoelectric point (pI) values vary from 5.87 to 8.73, while in cattle, they range from 5.88 to 8.82. Buffalo normally have all their CLCN proteins classified as acidic, with the exception of CLCN2, CLCN7, and CLC-KA, which are identical to cattle. Among the aliphatic index values of the buffalo and cow, the CLCN peptides are greater than 65, demonstrating their thermostability at elevated temperatures. That means that all the CLCN proteins have lower GRAVY values and are hydrophobic (Table 1b).

### 3.3. Structural Characterization

**Figure 2:**
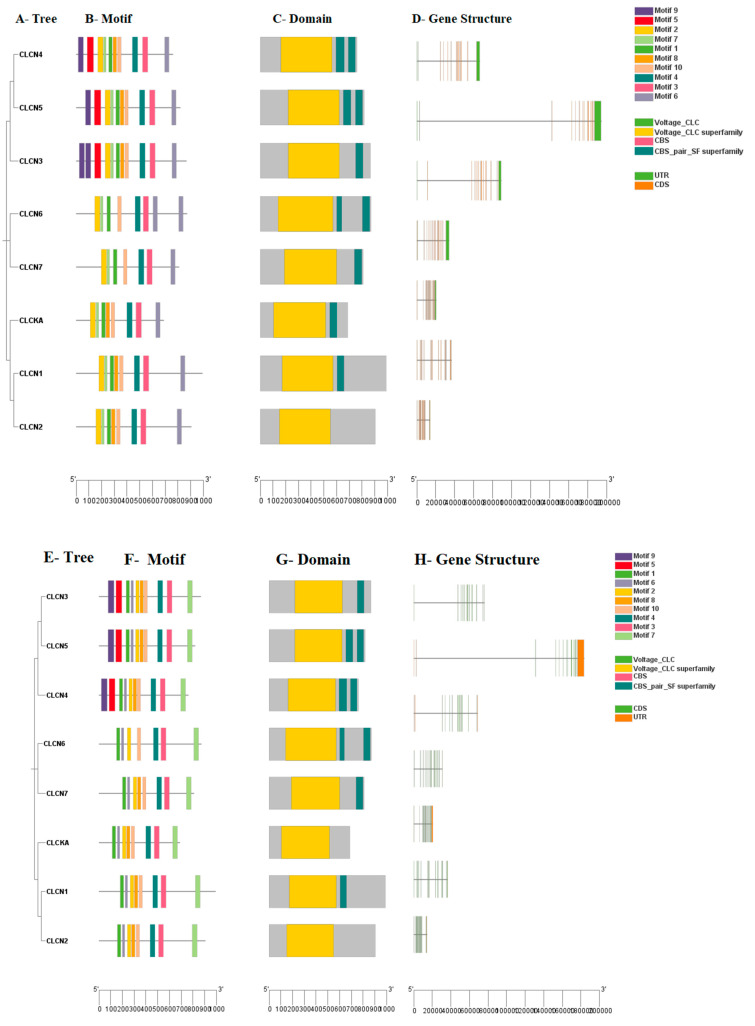
(**A**) Phylogenetic analysis of buffalo *CLCN* gene, (**B**) motif patterns, and (**C**) conserved domain of the buffalo *CLCN* genes. (**D**) Gene structure of buffalo *CLCN* gene family, (**E**) phylogenetic relationship of cattle *CLCN* gene, (**F**) motif patterns, and (**G**) conserved domain of the cattle *CLCN* genes. (**H**) Gene structure of cattle *CLCN* gene family.

**Figure 3:**
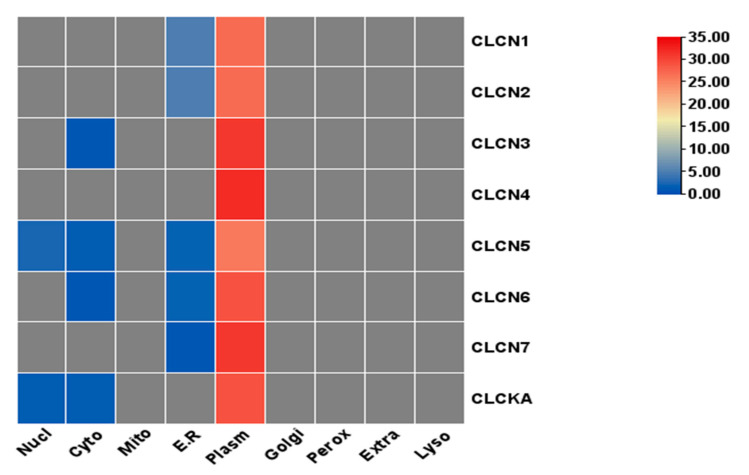
Distribution of *CLCN* gene distribution in different cells of buffalo.

The gene structure and conserved motifs of the cow and buffalo *CLCN* gene families were studied in order to get a deeper understanding of the buffalo *CLCN* gene family (Figure 2). The graphic or diagram displays the evolutionary relationship between the *CLCN* genes of cattle and buffalo (Figure 2A,E). For the *CLCN* gene family in cattle and buffalo, 10 conserved motifs were predicted (Figure 2B,F and Table 2). Pfam was further examined to predict the connections of these 10 conserved motifs with protein families. The Voltage_CLC domains were matched by the two, three, and four MEME motifs presented in buffalo (Supplementary Table S2a). Similarly, the Voltage_CLC domains were matched to MEME motifs three and four in cattle (Supplementary Table S2b). NCBI CDD searches were used to cross-confirm these domains (Figure 2C,G). Furthermore, a structural analysis demonstrated that the exon and intron patterns of the *CLCN* gene families in cattle and buffalo vary (Figure 2D,H). With the exception of CLCN3, CLCN4, and CLC-KA, further investigation on the subcellular localization of buffalo CLCN proteins showed that most of the proteins were expressed in the endoplasmic reticulum and plasma membrane (Figure 3).

### 3.4. Circos and Duplication Analysis

**Figure 4:**
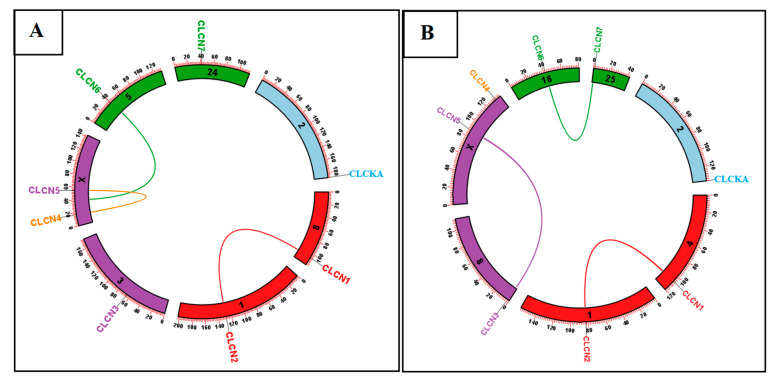
(**A**) Circos plot of *CLCN* genes in buffalo. (**B**) Circos plot of *CLCN* genes in cattle.

The precise placement of each gene on the chromosomes reveals the links between the *CLCN* gene pairs, which control the dimensions, positions, and orientations of the related genomic elements. The given data show the location and duplication events of the *CLCN* genes (Figure 4A,B). To understand more about the evolutionary history of the buffalo and cattle *CLCN* gene families, duplication occurrences were investigated. Tandem duplication of *CLCN4-CLCN5* was identified in buffalo, whereas three duplicated gene pairs were identified as segmental duplications (Table 2a,b). In order to determine the genomic areas with the ability to code for proteins, these gene pairs were also put through the Ka/Ks ratio test, which analyzes the divergence rates of synonymous and nonsynonymous sequences. For each pair of genes, the Ka/Ks test ratio findings revealed that none of the ratios was more than one, indicating that these proteins were subject to positive selection. We also showed that purifying selection was applied to every gene combination.

**Table 2:** (**a**) Ka/Ks ratio analysis of each gene pair in buffalo *CLCN* gene family. (**b**) Ka/Ks ratio analysis of each gene pair in cattle *CLCN* gene family.

Gene Pairs	Chromosomes	Duplications	Ka	Ks	Ka/Ks	Selection	Time (MYA)
			**(a)**	**Buffalo**			
*CLCN4*– *CLCN5*	X/X	TD	0.156732	2.767259	0.056638	Purifying selection	12.5
*CLCN6*– *CLCN7*	5/24	SD	0.540831	1.474348	0.366827	Purifying selection	6.70
*CLCN1*– *CLCN2*	8/1	SD	0.384904	1.511649	0.254625	Purifying selection	6.87
			**(b)**	**Cattle**			
*CLCN3*– *CLCN 5*	8/X	SD	0.196881	2.874906	0.068483	Purifying selection	13.1
*CLCN6*– *CLCN7*	16/25	SD	0.545979	1.516574	0.360008	Purifying selection	6.89
*CLCN1*– *CLCN2*	4/1	SD	0.362712	1.679889	0.215914	Purifying selection	7.63

SD: segmental duplication; Ka: nonsynonymous substitutions; Ks: synonymous substitutions; MYA: millions of years ago.

### 3.5. Scan PROSITE Analysis and Analysis of Syntenic Relationships

**Figure 5:**
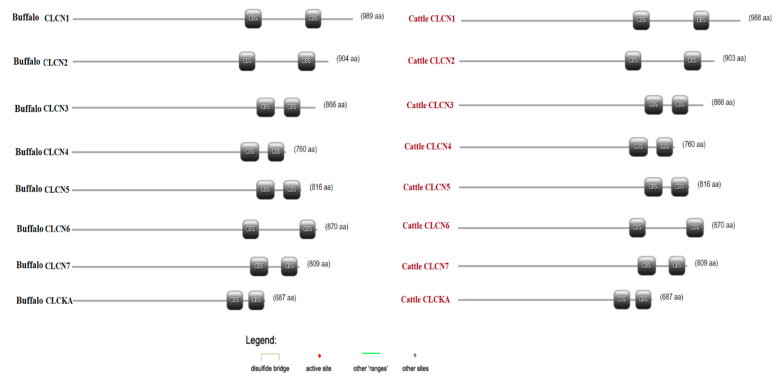
Intra-domain prediction with Scan PROSITE.

PROSITE was utilized to search for structural and functional residues linked to ProRule and the PROSITE signature in the CLCN proteins. Intra-domain characteristics including disulfide bridges, binding sites, and active sites are identified using this method. Combining motif recognition specificity and profile sensitivity improved the functional prediction accuracy. A single [CBS] hit was identified in both buffalo and cattle in (Figure 5), which shows a graphical representation of CLCN protein hits and feature predictions based on a Scan PROSITE database domain analysis. Genome conservation was observed by comparative genomic synteny analysis, which was carried out with the Circoletto tool. The synteny diagram demonstrates the important relationships in terms of the duplication, triplication, evolution, function, and expression between various species. The *CLCN* gene syntenic relationships between buffalo and cattle illustrate the special bond between cattle and buffalo (Supplementary Figure S9). The rate of conservation was represented by inward tangling ribbons with different color intensities in the comparative synteny analysis, whereas duplication events were shown by outward tangling ribbons. The genomic dynamicity and evolutionary alterations along mobile elements in the genomes of cattle and buffalo were ascertained using the syntenic rings. In the processes of chromosomal duplication, triplication, and rearranging, mobile components are essential. Blocks that take up permanent residence in the genome have the ability to cause alterations in expression, which may disrupt other biological pathways. Different colors indicate the similarities in both cattle and buffalo *CLCN* genes: the green color represents that the similarities between the buffalo sequence and cattle sequence are ≤0.50, the orange color is ≤0.75, and red is >0.75, respectively (Supplementary Figure S9).

### 3.6. Protein Structure Prediction

Three-dimensional structure model prediction (Supplementary Figure S10) and secondary structure prediction (Supplementary Table S3) were performed for the buffalo and cattle CLCN proteins. CLCN5 and CLC-KA, two related CLCN proteins located in cattle and buffalo, showed comparable amounts of secondary structural components such as β-helices and β-sheets as well as varying degrees of disorder.

### 3.7. Transcription Factor Binding Sites (TFBSs) Analysis

**Figure 6:**
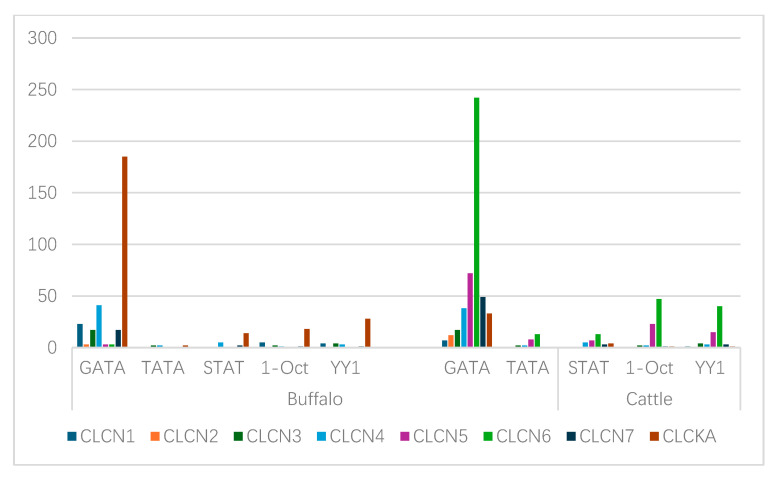
*CLCN* gene transcription factor binding sites (TFBSs) between buffalo and cattle.

The binding of transcription factors to certain genomic locations is the basis for transcriptional regulation in cells. Based on five transcription positions, the TATA, OCT1, GATA, YY1, and STAT transcription factor binding sites (TFBSs) in the *CLCN* gene family in cattle and buffalo were examined. The distribution pattern of the TFBSs within the CLCN gene family in buffalo was as follows: GATA > YY1 > OCT1 > STAT > TATA. The pattern seen in cattle was GATA > OCT1 > YY1 > STAT > TATA. All things considered, there were more TFBSs in cattle than in buffalo (Figure 6).

### 3.8. Recombination Analysis (GARD)

**Figure 7:**
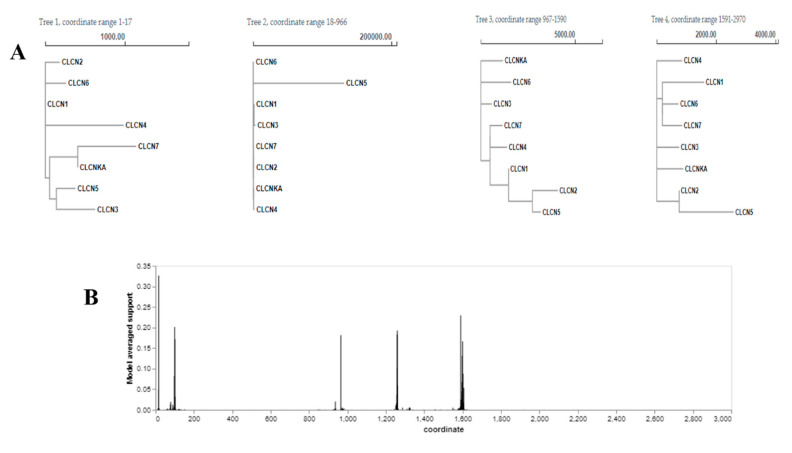
GARD analysis. (**A**) Trees for individual fragments. (**B**) Model-averaged support for breakpoint placement.

The recombination breakpoints identified by GARD were used to identify fragmented sequences, and the analyses of these sequences showed phylogenetic segregation across several recombination fragment trees: 1-17 (Tree 1), 18-966 (Tree 2), 967-1590 (Tree 3), and 1591-2970 (Tree 4) (Figure 7A). GARD examined 8824 models to identify evidence of recombination breakpoints, and 2965 putative breakpoints were located, with up to 3 inferred breakpoints. Remarkably, the genetic algorithm examined just 0.00% of these (Supplementary Figure S11). By measuring the frequency of detecting a breakpoint at a certain site over all the alignment points using the standardized Akaike weights of the models, the model-averaged support for the breakpoint sites was determined. The best-fitting model and this analysis was consistent. The multiple sequence alignment of eight *CLCN* gene nucleotide sequences, through multiple breakpoint studies with a genetic algorithm, revealed five main recombination breakpoints, with additional minor breakpoints at various places (Figure 7B).

## 4. Discussion

Advancements in next-generation sequencing and other high-throughput genome sequencing technologies have made it easier to screen for genetic diversity, such as SNPs, and their functional impact on certain phenotypic features. This skill makes it possible to comprehend animal genetics at the molecular level more thoroughly [30]. Candidate gene studies evaluate the genetic resources that are accessible for farm animals in order to predict functional genes and possible links between these genes and productivity attributes, including adaptability, disease resistance, and output capacity [43]. Comparative genomics provides the chance to investigate the genetics of physiological features that are commercially significant in buffalo by identifying new genes and the processes that govern them. The buffalo industry has benefited greatly from the research that has been conducted [44].

### 4.1. Phylogenetic Analysis

According to our results of the phylogenetic research, the buffalo *CLCN* gene family is closely linked to cattle and shares more sequence similarities with the Murrah buffalo and swamp buffalo. Furthermore, it was discovered that, although they have a clear evolutionary relationship with rat and mouse, Karan fries cattle are linked to *Bos taurus*, *Bos indicus*, and buffalo [45]. Furthermore, the overall phylogenetic connections showed that *Bos mutus*, *Bos taurus*, and *Bos indicus* are more closely linked to the buffalo (*Bubalus bubalis)* *CSN* gene family [44].

### 4.2. Physiochemical Properties of CLCN Gene Families

Determining the physicochemical characteristics of proteins belonging to different gene families is essential in comprehending their characteristics and roles. In the given species, the CLCN2 and CLCN-KA possess an acidic nature according to the isoelectric point (pI). The structure of an organism’s globular protein and its thermostability has been previously reported to be associated with the aliphatic index, AI; this index explains just how stable these proteins are at different temperatures [46]. The solvation of aliphatic side chains (leucine, isoleucine, valine, and alanine) to the volume of the protein is called the aliphatic index, AI, and is positive for increasing the thermostability of the globular protein. An organism can therefore be said to have a higher AI, if the selected protein is comparatively stable at higher temperatures [46]. Because the aliphatic index of buffalo and cattle CLCN peptides was greater than 65, it was concluded that both of them were thermostable. Because of the differences in their isoelectric point (pI) values, there were instabilities of which, out of the five proteins mentioned, few proteins clearly depicted instabilities. The grand average of hydropathicity, or the GRAVY value, is used to forecast how proteins and water would interact. It is determined by dividing the total hydropathy values of a protein by the length of the protein. Quantifying a protein’s hydrophobic or hydrophilic characteristics is made easier with the GRAVY score. A protein with a negative GRAVY score is considered to be hydrophilic, whereas a positive GRAVY value denotes a hydrophobic protein [47]. The lower GRAVY evaluations of all the CLCN proteins revealed that they were hydrophobic.

### 4.3. Sequence Analysis

Protein sequence data can highlight significant evolutionary-conserved regions that are essential for a variety of biological processes. One important technique for locating these conserved regions and gathering information required for the structural and functional investigation of proteins is multiple sequence alignment [48]. Using the MEME tool, ten buffalo *CLCN* gene family motifs were predicted in order to study the protein sequence properties of the CLCN proteins. Information is provided on the regular expression levels of these conserved motifs. The structural and functional arrangement of the proteins depends on these common motifs. According to our research, the Voltage_CLC domains of both species include three and four MEME motifs, respectively. A similar study has also employed these motif predictions to investigate biological roles and conserved limitations in several animal genes [32].

### 4.4. Gene Duplication Analysis

In order to acquire novel genes or genetic variations, organisms employ a variety of gene duplication mechanisms, including crossing over, retro-position, and genome or chromosomal duplication. Through the creation of genetic variation and the facilitation of the formation of novel or enhanced functions, these mechanisms play a major role in the evolution of functional processes [49]. It is important to recognize the dynamics of gene duplication and the trajectories of duplicated genes that follow, because previous studies provide insights on the features of evolutionary forces that are both localized and genome-wide. Previous studies also reported on the linkages and interactions that influence genetic diversity and adaptability during the evolutionary process, as well as the evolution of intra-specific and interspecific genome contents [49]. Although measuring the pace of gene duplications can be difficult, the emergence of redundant genetic variations is mostly driven by selection pressure and mutations with functional implications. Thus, as these variables influence the retention of duplicated genes and their divergence and capability to acquire new roles, they influence the evolution of those genes. In the same number of generations, it is easier for a duplicated gene to transmit undesired changes as compared to transmitting an effectively working copy of the gene. This enhanced rate of mutation might make a gene perform other tasks or be more intricate in the biological procedures than its original counterparts and lead to the introduction of new functions or adaptations [50]. According to previous studies on ice fish, apparent defects in a duplicated digestive gene result in the antifreeze gene, whereas duplication provides a different gene for snake venom [50], and in pigs, it results in the synthesis of 1-β-hydroxytestosterone [51]. Thus, to elucidate the evolutionary history of buffalo and cattle on *CLCN* gene families, the investigation examined duplication events on these gene families. The three segmental duplications of the duplicate gene pairs were identified, and a particular buffalo-specific tandem duplication in *CLCN4* and *CLCN5* was also evident. The Ka/Ks ratio test was then used to compare the rate of nonsynonymous (Ka) and synonymous (Ks) changes in these gene pairs offering insight to the selection forces acting on these genes and their possible functional divergence. All the observed ratios did not exceed one, based on the Ka/Ks ratio test, thus implying that these gene pair’s proteins are under purely selective constraints. This means that although this study is implying that the genes are slowly evolving, there is a functional constraint that ensures that the basic roles of those genes are maintained. Their functional integrity is being maintained in cattle and buffalo alike, despite the existence of gene duplications.

### 4.5. Scan PROSITE Analysis and Analysis of Syntenic Relationship

Using the PROSITE analysis, the particular residues that play a role in the CLCN protein activity and interactions such as the disulfide bridge, active site, or binding site and the structure function correlation remain to be defined. When compared to the above predictions, this work improves the results of the sensitivity of the profile and the specificity of finding motifs with the ProRule and PROSITE signature. Likewise, similar methodologies were used in other research studies, such as the one that used *Bufo bufo* to determine the key features of protein and their impact on organisms. The synteny diagram presents an outstanding relationship among these species regarding duplication, triplication, function, expression, and evolution, which depicted an exclusive relationship between buffalo and cattle. The findings of this study provide for the existence of, in fact, a quite limited number of syntenic relations and conserved genomic segments in the species included in the given analysis. This indicates how the evolutionary events and conserved genes have worked on the genomics and functional domains of buffalo and cattle. They also support the observation of these processes as identified by [38].

### 4.6. Protein Structural Configuration

Eukaryotic proteins have a modular structure with an N-terminal ATP binding domain connected to a middle domain by a “linker” of variable length. The N-terminal and middle domains together form a “split” ATPase site that is also the binding pocket for GA. The three-dimensional structures and configurations of the CLCN proteins in buffalo and cattle were evaluated. The analysis of 3-D secondary protein structures indicated that the structural variations were present only in CLCN3 and CLCN-KA of buffalo and cattle, whereas other CLCN genes were structurally related in both species. These findings verify the outcomes from the phylogenetic and gene structural analyses, which show that CLCN proteins are conserved. As chaperones, CLCN3 and CLCN-KA are essential for protein folding in cells. There were differences in the total amount of amino acid residues and secondary structural characteristics between the three-dimensional structures of CLCN3 and CLCN-KA in cattle and buffalo. These variations could be related to the unique roles that chaperones play and the cellular reactions that are observed in every species. To verify these results, a further transcriptome-level study is required [52].

### 4.7. Transcription Binding Sites

Based on earlier findings, the YY1 repressor site and transcriptional binding sites (*STAT*, *GATA*, *TATA*, and *OCT1*) were present in this study’s analysis of the genomic sequences of buffalo and cattle. OCT1, which is well known for its involvement in acute myeloid leukemia (AML), shows less inhibition of DNA binding and increases *CSN* gene expression. After becoming phosphorylated and dimerized, STAT moves into the nucleus, attaches itself to DNA, and increases transcription [33]. YY1 uses a variety of methods to suppress transcription. The transcriptional repression of genes is usually caused by YY1 binding to particular locations on DNA and interacting with activating signals [53]. YY1 and CREB often act jointly in the nucleus to inhibit transcription [33]. Consequently, to increase the repression activity of YY1, cofactor interactions such as those with mRPD3 or other family members are usually necessary [54]. Specific examination of the regulation of the *CLCN* gene family shows in detail that transistor perception on certain sites of the genome is very important. The present analysis also shows that cattle have more transcription factor binding sites (TFBSs) than buffalo.

### 4.8. Recombination Analysis Using Genetic Algorithm Recombination Detection (GARD)

In the present study, five significant potential breakpoints in the nucleotide sequences of *CLCN* genes were identified by recombination analysis using GARD. Many roles that the *CLCN* gene family plays in various animals are facilitated by these breakpoints. Variations in these sequences are driven by evolutionary forces and result in the observed functional diversity [41]. It is suggested that at least one of the breakpoints represents a topological incongruence when comparing the model that assumes a uniform tree for all partitions but allows for various branch lengths with the Akaike Information Criterion (AIC) scores, which allow for different topologies between segments. Examining this variation may give light on the underlying biological or evolutionary processes that developed it, or it may highlight certain traits of the species tree [55]. It is also critical to investigate this problem within a methodological framework, taking into account the potential impact of phylogenetic inference errors or uncertainties on the observed topological incongruence between gene trees and the species tree [56].

## 5. Study Limitations

The in silico investigations on the molecular characterization of the *CLCN* gene family by using a genome-wide analysis offer valuable insights into the genomic landscape and the functional significance and precise roles of the identified *CLCN* genes. However, it is crucial to acknowledge several limitations that are inherent to this study. First, issues with gene annotation might potentially exist; errors or omissions in the annotation procedure could impact the accuracy of the outcomes. Moreover, conducting functional validation of the predicted *CLCN* genes is vital for a comprehensive understanding of their biological significance. Second, a lack of experimental validation might be a significant limitation. Additionally, technical constraints associated to bioinformatics tools and algorithms may introduce biases or inaccuracies into the results of this study. By addressing these constraints in further research initiatives, it is strongly probable that deeper insights and more credibility of the findings would enhance the understanding of the *CLCN* gene family in buffalo as well as other species.

## 6. Conclusions

Our study provides in depth knowledge about the *CLCN* gene family that has been conserved throughout evolution, and buffalo showed more structural and sequencing similarities with cattle. Eight *CLCN* genes were identified and divided into three subfamilies according to the phylogenetic study. A conserved pattern of genomic blocks between buffalo and cattle has been confirmed by a synteny analysis. Due to a change in the structural residues of *CLCN2* and *CLCN-KA*, this may impact on protein functioning. Additionally, transcription binding sites might have an impact on buffalo characteristics including body weight, height, and muscular growth and have an influence on milk traits. Moreover, five major recombination breakpoints present in *CLCN* buffalo genes and variations in these sequences are driven by evolutionary forces, and it results in functional diversity. Our studies would aid in a better understanding and critical function of *CLCN* genes and their possible application in selective breeding of buffalo for economically significant qualities, including reproduction, growth, and development.

## Figures and Tables

**Table 1:** (**a**) Physiochemical properties of buffalo and cattle *CLCN* gene family. (**b**) Physiochemical properties of buffalo and cattle *CLCN* gene family.

Genes	Chr	AA Length	Molecular Weight (kDa)	Theoretical pI	Instability Index	Aliphatic Index	Gravy
			**(a)**	**Buffalo**			
*CLCN1*	8	989	109.02	5.87	57.50	92.86	0.093
*CLCN2*	1	904	99.35	8.72	53.72	93.25	0.092
*CLCN3*	3	866	96.20	5.94	38.72	93.93	0.110
*CLCN4*	X	760	84.89	6.39	39.13	99.80	0.221
*CLCN5*	X	815	90.72	6.05	38.54	98.46	0.203
*CLCN6*	5	870	97.19	6.52	44.06	93.07	0.118
*CLCN7*	24	809	88.78	7.97	37.85	102.92	0.277
*CLCNKA*	2	687	75.18	8.73	38.03	98.22	0.373
			**(b)**	**Cattle**			
*CLCN1*	4	988	108.85	5.88	57.45	91.97	0.075
*CLCN2*	1	903	99.21	8.78	52.27	93.24	0.086
*CLCN3*	8	866	96.14	5.94	38.57	93.59	0.108
*CLCN4*	X	760	84.89	6.39	39.13	99.80	0.221
*CLCN5*	X	816	90.72	6.05	38.54	98.46	0.203
*CLCN6*	16	870	97.23	6.52	43.96	92.95	0.116
*CLCN7*	25	809	88.83	7.97	37.86	103.63	0.289
*CLCNKA*	2	687	75.17	8.82	38.44	98.66	0.361

## Data Availability

The data supporting this study’s findings are included in the manuscript or the Supplementary Information.

## Supplementary Materials

The following supporting information can be downloaded at: https://www.mdpi.com/article/10.3390/genes15091163/s1. Table S1: *CLCN* genes in buffalo and cattle. Table S2: a: Ten significantly conserved motifs within the buffalo *CLCN* gene family. b: Ten significantly conserved motifs within the cattle CLCN *gene* family. Table S3: CLCN protein secondary structure features predicted. Figure S1. Multiple sequence alignment *CLCN1*. Figure S2. Multiple sequence alignment *CLCN2*. Figure S3. Multiple sequence alignment *CLCN3*. Figure S4. Multiple sequence alignment *CLCN4*. Figure S5. Multiple sequence alignment *CLCN5*. Figure S6. Multiple sequence alignment *CLCN6*. Figure S7. Multiple sequence alignment *CLCN7*. Figure S8. Multiple sequence alignment *CLCN-KA*. Figure S9: Analysis of *CLCN* gene syntenic relationships between buffalo and cattle. Figure S10: Tertiary structures of CLCN protein in buffalo and cattle; blue color showed N-terminus, red color showed C-terminus, green color showed coils and loops, yellow color showed beta sheets. Figure S11. GARD analysis.
