# Supplementary material for: In Silico Analysis: Molecular Characterization and Evolutionary Study of CLCN Gene Family in Buffalo
Source: Genes (Basel). 2024 Sep 3;15(9):1163. doi: 10.3390/genes15091163 (PMC11431104; doi:10.3390/genes15091163)
Supplement: Supplementary file 1 [file genes-15-01163-s001.zip › genes-3174556-supplementary.pdf]

Supplementary Data

Supplementary Table S1: CLCN genes in buffalo and cattle

Specie	Genes	Protein ID	CDS ID	Source
Buffalo	CLCN1	NP_001277845.1	NM_001290916.1	NCBI
	CLCN2	XP_025143117.3	XM_025287332.3	NCBI
	CLCN3	XP_045020911.1	XM_045164976.1	NCBI
	CLCN4	XP_006050752.1	XM_006050690.4	NCBI
	CLCN5	XP_006059537.1	XM_006059475.4	NCBI
	CLCN6	XP_045021658.1	XM_045165723.1	NCBI
	CLCN7	XP_025131357.1	XM_025275572.2	NCBI
	CLCKA	XP_006066655.2	XM_006066593.3	NCBI
Cattle				
	CLCN1	NP_001137343.1	NM_001143871.1	NCBI
	CLCN2	NP_001095957.1	NM_001102487.1	NCBI
	CLCN3	NP_001179932.1	NM_001193003.1	NCBI
	CLCN4	NP_001026927.1	NM_001031757.1	NCBI
	CLCN5	XP_005228237.1	XM_005228180.5	NCBI
	CLCN6	NP_001192799.1	NM_001205870.1	NCBI
	CLCN7	NP_001020502.1	NM_001025331.1	NCBI
	CLCKA	XP_024839117.1	XM_024983349.2	NCBI

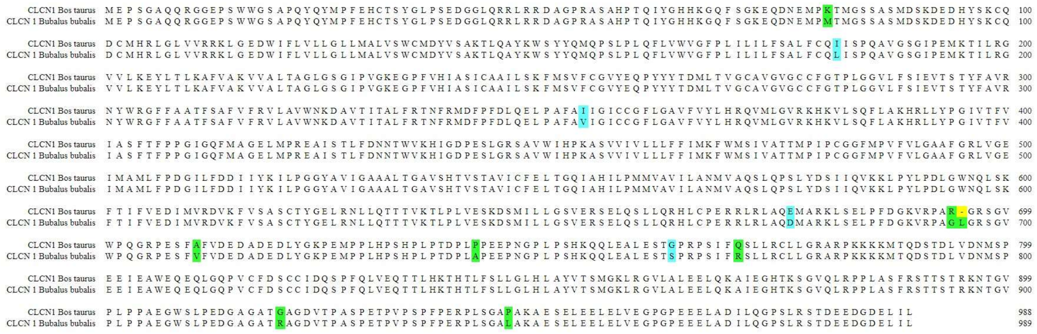

Supplementary Figure S1. Multiple Sequence Alignment CLCN1

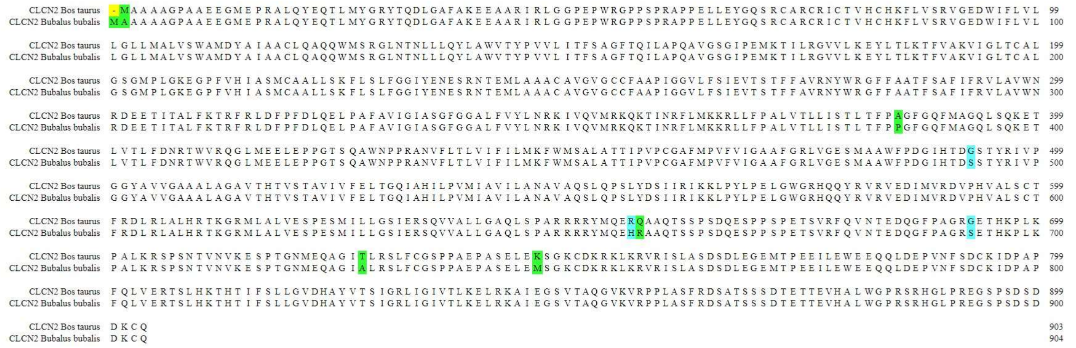

Supplementary Figure S2. Multiple Sequence Alignment CLCN2

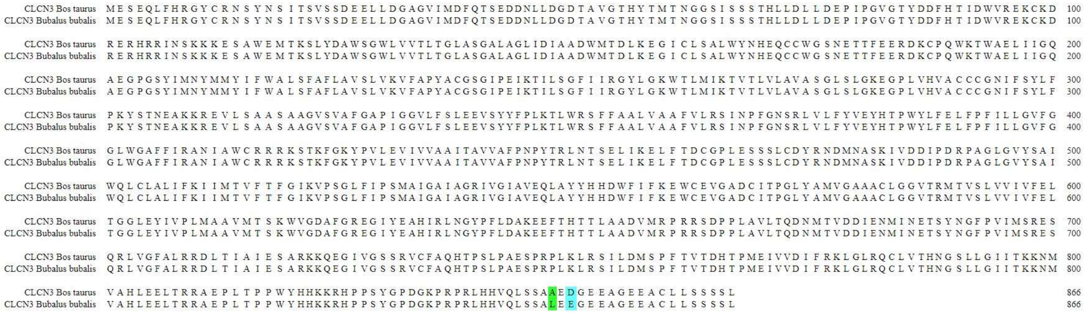

Supplementary Figure S3. Multiple Sequence Alignment CLCN3

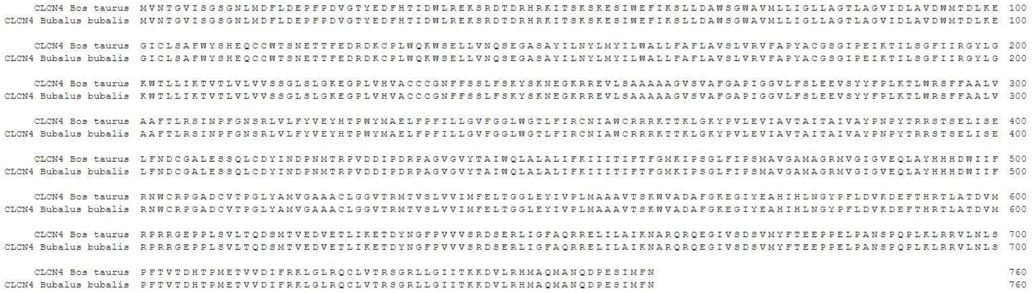

Supplementary Figure S4. Multiple Sequence Alignment CLCN4

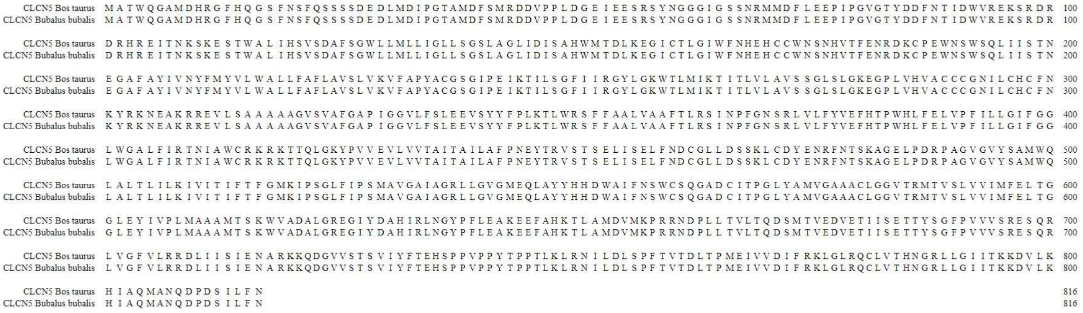

Supplementary Figure S5. Multiple Sequence Alignment CLCN5

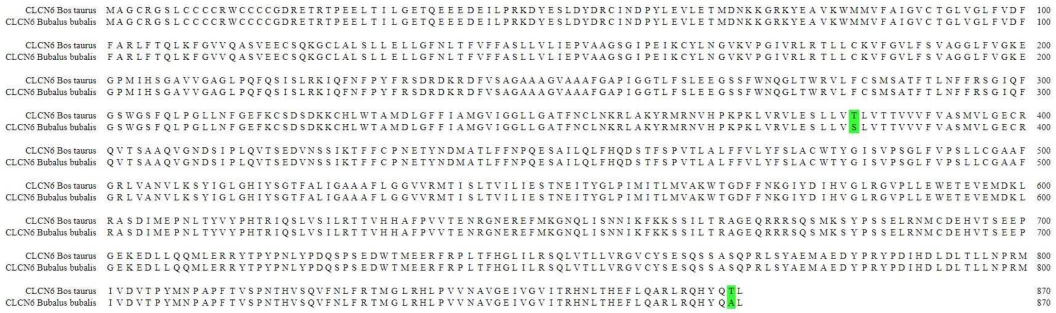

Supplementary Figure S6. Multiple Sequence Alignment CLCN6

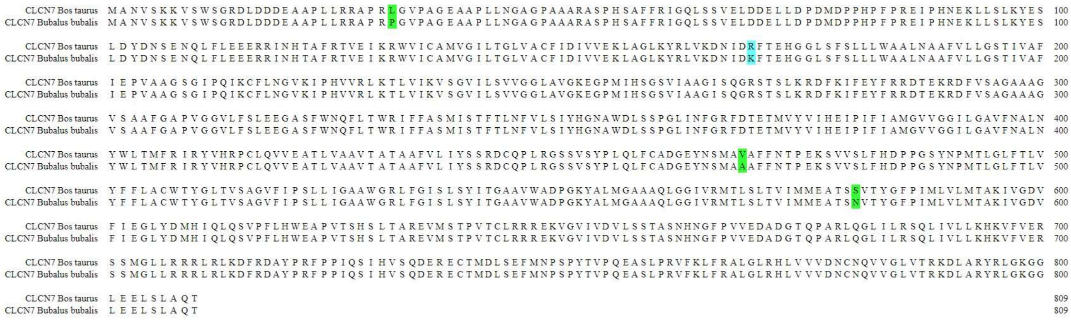

Supplementary Figure S7. Multiple Sequence Alignment CLCN7

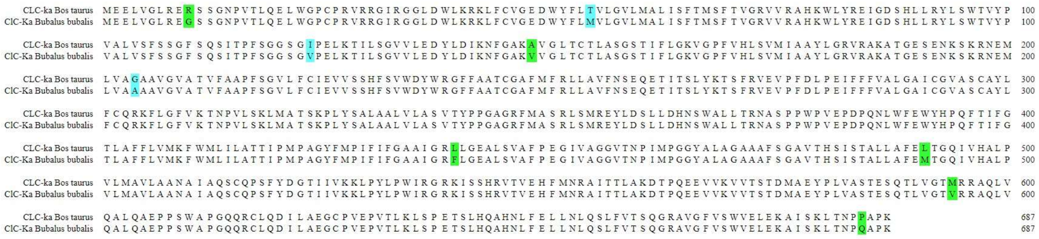

Supplementary Figure 8S. Multiple Sequence Alignment CLCN-KA

Supplementary Table S2a: Ten significantly conserved-motifs within the Buffalo CLCN gene family.

MEME Motif	Sequence of amino acid	Width	Pfam Domain
1	SAAAAAGVSVAFGAPIGGVLFSJEEVSSY	29	-

2	QVIAPYACGSGIPEIKTILSGVIIRGYLGLKTLVIKVVGLV	41	Voltage_CLC
3	AALGGVVRMTVSLAVIMFELTGGIEYILPLMAAVMTAK WVAD	42	Voltage_CLC
4	TLALFLIMKFWMTIFTFGIPIPSGLFIPSMAIGAAFGRLVG	41	Voltage_CLC
5	IDIAADWMTDLKEGICLSALWYNHEQCCWGSNETTFED RDKCPQWKSWSE	50	-
6	LDPSPFTVTEHTPLEKVVDJFRKLGLRHLLVTSN	34	-
7	SGLSLGKEGPLVHVASCCGA	20	-
8	LWRGFFAALVAAFVLRSJAPFGNSRLVJT	29	-
9	DEPIPGVGTYDDFHTIDWVREKSRDRDRHRKITSKSKES AW	41	-
10	PWDLQELPPFILLGVFGGLWGALFIYLNR	29	-

Supplementary Table S2b: Ten significantly conserved-motifs within the Cattle CLCN gene family.

MEME Motif	Sequence of amino acid	Width	Pfam Domain
1	APYACGSGIPEIKTILSGVIIRGYLGLKT	29	-
2	SAGAAAGVSVAFGAPIGGVLFSJEEVSSY	29	-
3	AALGGVVRMTVSLAVIMFELTGGIEYILPLMAAVMTA KWVAD	42	Voltage_CLC
4	TLALFLIMKFWMTIFTFGIPIPSGLFIPSMAIGAAFGRLV G	41	Voltage_CLC
5	IDIAADWMTDLKEGICLSALWYNHEQCCWGSNETTFE DRDKCPQWKSWSE	50	-
6	SGLSLGKEGPLVHVASCCGA	20	-
7	LDPSPFTVTEHTPLEKVVDJFRKLGLRHLLVTSNGRLIG II	41	-
8	WRGFFAALVAAFVLRSJAPFGNSRLVJTY	29	-

9	PGVGTYDDFHTIDWVREKSRDRDRHRKITSKSKESAW ELIKSLYDAWSGW	50	-
10	PWDLQELPPFILLGVFGGLWGALFIYLNR	29	

Supplementary Table S3: CLCN Proteins secondary structure features predicted

Specie				
Buffalo	Protein	Alpha helix	Beta Strand	Disorder
	CLCN1	42%	3%	32%
	CLCN2	43%	3%	28%
	CLCN3	47%	4%	14%
	CLCN4	49%	4%	14%
	CLCN5	49%	4%	14%
	CLCN6	44%	3%	16%
	CLCN7	44%	4%	16%
	CLCKA	51%	5%	6%
Cattle				
	CLCN1	42%	4%	31%
	CLCN2	43%	3%	25%
	CLCN3	47%	4%	16%
	CLCN4	52%	4%	6%
	CLCN5	49%	4%	14%
	CLCN6	44%	3%	14%
	CLCN7	45%	3%	16%
	CLCKA	51%	5%	6%

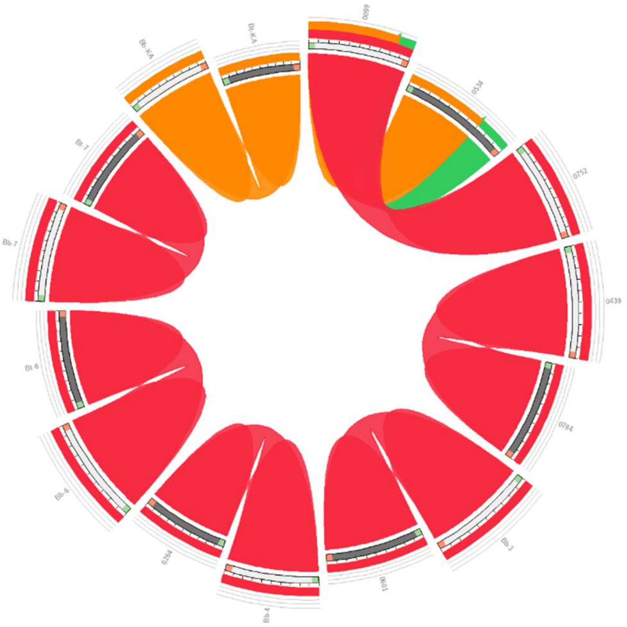

Supplementary Figure S9: Analysis of CLCN gene syntenic relationships between buffalo and cattle

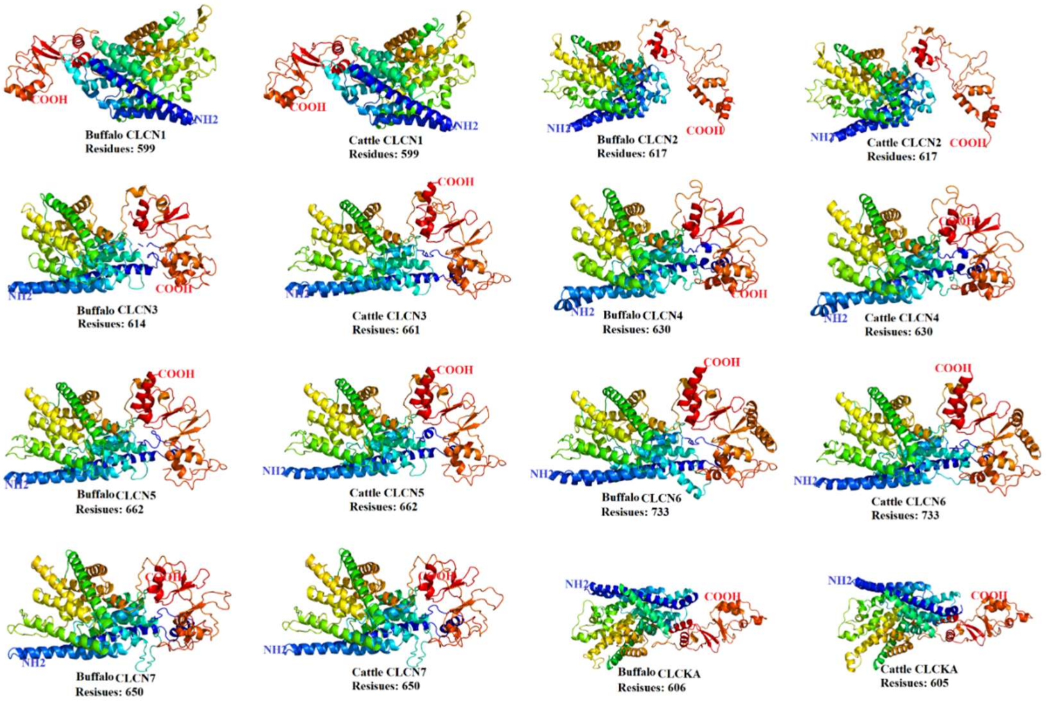

Supplementary Figure S10: Tertiary structures of CLCN protein in buffalo and cattle, blue color showed N-terminus, red color showed C-terminus, green color showed coils

and loops, yellow color showed beta sheets.

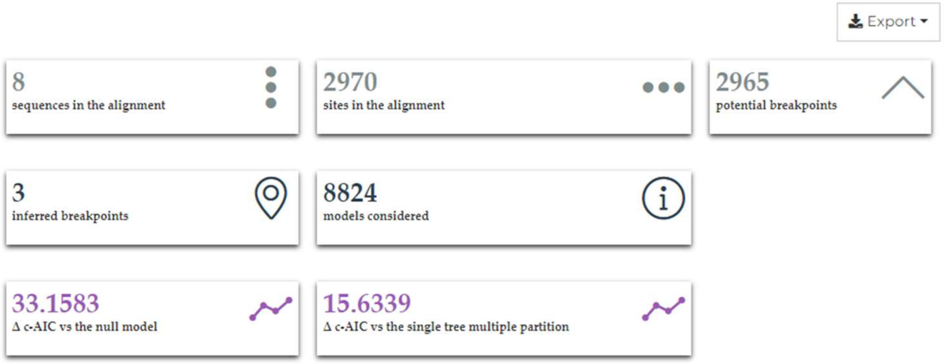

Supplementary Figure S11. GARD Analysis
